# Editorial: Person-Centred Rehabilitation – Theory, Practice, and Research

**DOI:** 10.3389/fresc.2022.980314

**Published:** 2022-07-20

**Authors:** Jacqui H. Morris, Nicola Kayes, Brendan McCormack

**Affiliations:** ^1^School of Health Sciences, University of Dundee, Dundee, United Kingdom; ^2^Centre for Person Centered Research, Auckland University of Technology, Auckland, New Zealand; ^3^Faculty of Medicine and Health, Susan Wakil School of Nursing and Midwifery, The University of Sydney, Sydney, NSW, Australia

**Keywords:** person-centeredness, rehabilitation goals, person-centered practice, person-centered rehabilitation, person-centered framework

Person-centeredness in healthcare is a philosophical approach in which decision-making and caregiving is explicitly undertaken with, and not for the person, with their needs, values and preferences positioned as central to the care they receive (1). Person-centeredness recognizes and respects the personhood of individuals within their own contexts and is underpinned by the values of dignity, respect, compassion, curiosity, personalisation and supportive relationships (2). Within rehabilitation, person-centeredness is frequently recommended as a philosophical approach, however, research into its conceptualization and implementation within rehabilitation practice is relatively underdeveloped compared to other healthcare fields (2).

Within this topic in Frontiers in Rehabilitation Science, we present a collection of papers that address some of the key issues in this emerging academic and practice-orientated field. Attard et al. contribute a discussion paper presenting a novel perspective to the conceptualization of person-centeredness relevant to rehabilitation and recovery. They illustrate how the concepts of SECI and Ba from Japanese epistemological traditions can provide novel processes that could facilitate children's involvement in decision-making about their care, rehabilitation and recovery within child and adolescent mental health services. They propose that creating opportunities within the system (Ba) for eliciting and integrating the tacit and explicit knowledge of stakeholders using the SECI model will provide platforms for developing person-centered practices to ensure that the values, beliefs and experiences of parents, children and staff are heard and given equal value within the system. Curnow et al. draw on another conceptual framework—the Person-Centered Practice Framework (1) to explore how people experienced healthcare support during the COVID-19 pandemic. Using the framework as a lens, the authors evaluated how and why many experiences of accessing and receiving care, particularly for long-COVID, were negative. They also highlighted meaningful learning from positive experiences characterized by shared problem solving and acknowledging people as experts in their own condition. These papers illustrate the value of using theoretically informed frameworks to enhance our understanding of person-centered rehabilitation and care.

Person-centered goal-setting, in which meaningful goals are negotiated with recipients of rehabilitation and plans to achieve them are agreed upon, is considered fundamental to rehabilitation practice (3). Despite the espoused centrality of collaborative goal-setting to rehabilitation, clinicians, rather than the people with whom they work, often drive goal-setting, leading to poorer engagement and outcomes for recipients (3). In their article, Leeson et al. explored how clinicians worked with people with traumatic brain injury to identify a goal focus for a community-based social activity within the context of a community connection intervention. They identified humanizing, empowering, and focusing as key strategies for building a relational approach to underpin their intervention, concepts with broad application to goal-setting within many rehabilitation contexts. Building on the importance of context to person-centered rehabilitation, Eggen and Thuesen interviewed eight people with rheumatic diseases about the changing relevance of goals and plans made within in-patient rehabilitation to their lives after discharge. They found that goals and plans made within rehabilitation were acontextual to their lives after discharge, with being back home described as a harsh reality. Consistent with recent person-centered models for rehabilitation (2) and care (1), the study emphasizes that understanding the person's context in its physical, social and psychological entirety is vital to ensure congruence of goals and plans across care transitions.

The development and testing of person-centered interventions is another indication of the growth of the field within rehabilitation. The final paper within this collection by Wadams et al. is a systematic review of metacognitive treatment in acquired brain injury, explicitly evaluating its relevance to aphasia. Metacognitive therapy seeks to promote self-awareness and awareness of performance errors, thereby enhancing independence in goal setting and achievement, therapy participation and functioning. The review showed the intervention was effective across a range of outcomes, but the small number of studies examining its specific use in people with aphasia meant that it was impossible to determine effectiveness for that population, and more research is necessary. Metacognitive therapy is inherently person-centered and focuses on the individual and their specific goals and challenges. This important review provides another example of the expansion but relative immaturity of the field of person-centered rehabilitation.

Together these articles provide a snapshot of issues relevant within this growth area of research in rehabilitation. They provide a small but essential step in advancing knowledge to address some of the most challenging topics and in moving the almost universal rhetoric about person-centered rehabilitation within the field toward a steadfast reality.

## Author contributions

JM took the lead in writing the editorial. NK and BM reviewed drafts and provided critical feedback. All authors contributed to the article and approved the submitted version.

## Conflict of interest

The authors declare that the research was conducted in the absence of any commercial or financial relationships that could be construed as a potential conflict of interest.

## Publisher's note

All claims expressed in this article are solely those of the authors and do not necessarily represent those of their affiliated organizations, or those of the publisher, the editors and the reviewers. Any product that may be evaluated in this article, or claim that may be made by its manufacturer, is not guaranteed or endorsed by the publisher.
